# Simultaneous and rapid colorimetric detection of distinct miRNAs using Split-LAMP

**DOI:** 10.3389/fbioe.2023.1271297

**Published:** 2023-11-02

**Authors:** Yi Jing Chua, Steven Poh Chuen Sim, Medha Shridharan, Yiqi Seow

**Affiliations:** ^1^ Genome Institute of Singapore (GIS), Agency for Science, Technology and Research (A*STAR), Singapore, Singapore; ^2^ Institute of Molecular and Cell Biology, A*STAR (Agency for Science, Technology and Research), Singapore, Singapore; ^3^ NUS High School of Mathematics and Science, Clementi, Singapore

**Keywords:** multiplex miRNA, LAMP, POCT, split-LAMP, rapid colorimetric detection

## Abstract

**Introduction:** Aberrant microRNA (miRNA) expressions are often discovered in many life threatening diseases such as cancer. In particular, recent studies show combinations of miRNA levels have greater diagnostic accuracy as opposed to single miRNA levels. For point-of-care applications, rapid and sensitive isothermal amplification with loop-mediated isothermal amplification (LAMP) has gained significant interest.

**Method:** We developed a cost-effective point-of-care testing (POCT) device for multiple miRNAs that can integrate miRNA signals into a single output.

**Results and Discussion:** We demonstrate that the loop primers for LAMP can be broken and be used for miRNA detection. This split-LAMP approach provides a logic AND-gate output for two distinct miRNA inputs. We then show that this is potentially useable in point-of-care testing using pH-sensitive dye to give a rapid, colorimetric endpoint readout within 30 min. This novel logic gate approach can potentially be extended to multiple miRNAs such that there can be a powerful diagnostic concept for multiple short RNAs in a point-of-care rapid test.

## 1 Introduction

miRNAs are post transcriptional gene regulators of more than 60% of human protein-coding genes (Friedman et al., 2009; Jet et al., 2021). They belong to a class of short non-coding RNAs that are small yet closely intertwined with many biological and pathological processes (Han Li and Chen, 2015). In recent studies, miRNAs are identified as essential and potential biomarkers for various diseases such as cancers, cardiovascular diseases, and viral infection (Abdallah et al., 2023; Aquino et al., 2023; Ullah et al., 2023). The diagnosis of these diseases typically requires detection for multiple miRNAs as single miRNA levels are usually more variable, thus combinations possess higher accuracy and are more predictive in diagnostics as compared to single miRNA levels (Zhou et al., 2015; Chen et al., 2021). Therefore, there is great significance in improving methods for multiple miRNAs detection that could potentially lead to unprecedented developments in early clinical diagnosis.

Traditional methods for miRNA detection, such as RT-qPCR, Northern blotting, and microarray, have been widely used but faced challenges including long processing times, labor-intensive methods, cost, and sensitivity issues (Ye et al., 2019). Alternative miRNA detection approaches based on isothermal amplification, such as LAMP, have emerged because of their greater compatibility with point-of-care testing (POCT) (Moehling et al., 2021). LAMP-based methods also display a higher specificity due to the utilization of four to six primers to detect six to eight target sequences at the same time (Becherer et al., 2020). Recent technologies have enhanced LAMP-based detection. An example is Rolling circle and loop mediated isothermal amplification (RCA-LAMP) which couples rolling circle amplification with LAMP and has shown better sensitivity compared to RC-qPCR (Tian et al., 2019). miRNA reverse transcription-based LAMP assay uses phosphorothioated-modified terminal hairpin formation and self-priming (THSP) for initiating self-folding and DNA extension in the presence of target miRNA, offering better specificity (Abdullah Al-Maskri et al., 2020). However, most of these LAMP technologies have only been developed to detect single miRNA levels and are highly complex. Numerous studies have identified panels of miRNAs as promising biomarkers for many diseases, including gastric cancer (So et al., 2021). Thus, there is a need to develop novel technology to simultaneously detect multiple miRNAs via POCT for early clinical diagnosis.

We aim to explore the feasibility of developing a simplified and efficient LAMP assay for simultaneous multiple miRNAs detection, that is, suited for POCT. Hence, we seek to incorporate a logic AND logic gate concept to LAMP in order to detect the presence or absence of two miRNAs rather than a single miRNA as a single output, as none of the aforementioned LAMP methods have developed integration for miRNA biomarkers at the molecular level.

Conventionally, LAMP is an isothermal amplification method that relies on strand displacement to create single stranded loops, enabling primer binding and subsequent exponential amplification (Notomi et al., 2000). The key primers required for LAMP reaction are Forward Internal primer (FIP) and Backwards Internal Primer (BIP). In FIP, the F2 sequence is concatemerized with the reverse complement of the F1 sequence (F1c) and likewise in BIP, B2 is concatemerized with B1c. FIP first binds to the linear template and extends by DNA replication. The F3 primer displaces the FIP amplified strand from the double stranded DNA, allowing a loop to be formed when F1 binds to the F1c sequence. Likewise, the same happens with B3 and BIP. Amplification results from FIP/BIP binding onto the loop structure and causes branching of the DNA strand to form more loops. To speed up the LAMP reactions, Loop primer F (LF) and Loop primer B (LB) are sometimes used to open the reverse complement loop structure for more branching and loop formation.

However, the use of traditional LAMP to develop a miRNA LAMP-based assay is challenging as miRNAs are about 22 nucleotides in length (Felekkis et al., 2010), thus are too short to be used as templates in traditional LAMP. Additionally, it is also not practical to utilize the entire sequence of FIP and BIP as analytes, as these primers are usually composed of over 40 nucleotides which exceeds the length of miRNAs. New approaches are required to overcome these difficulties in the development of a miRNA LAMP-based assay. Here, we demonstrated a functional, colorimetric Split-LAMP assay which displays a single diagnostic readout (<30 min), that is, positive only when two distinct miRNAs are detected simultaneously. This AND gate concept is incorporated with the assay by using miRNAs as primers, rather than templates, in amplification. This is achieved by splitting FIP and BIP into their constituent primers, namely, F1c and F2 for FIP, and B1c and B2 for BIP. The F2 and B2 primers are then replaced by miRNA analytes that are detected for diagnostics.

## 2 Materials and methods

### 2.1 Materials and reagents

Split LAMP primers and oligonucleotides shown in Table 1 were purchased from Integrated DNA Technologies (IDT). The HEK-293 cell line (catalog number CRL-1573, American Type Culture Collection) (ATCC) was used in this study.

**TABLE 1:** Primers and oligonucleotides used.

Primer and oligonucleotides	Sequence (5′ 3′)
Template Ultramer (double loop structure)	CGG​AGA​GGT​CGC​GAT​AGT​CAT​GCT​TAT​CAG​ACT​GAT​GTT​GAT​GGT​CTA​CGG​CCA​GAT​CAG​TGA​CTG​ACT​ATC​GCG​ACC​TCT​CCG​GTG​ATG​ACA​GTG​ACA​TCC​TGC​CTG​TGA​CAG​GAC​ATC​GGT​GAC​AGT​TAC​AAC​CAG​CTA​AGA​CAC​TGC​CTA​GGC​AGG​ATG​TCA​CTG​TCA​TC
LF	TCA​CTG​ATC​TGG​CCG​TAG​ACC​A
LB	TGA​CAG​GAC​ATC​GGT​GAC​AGT
FIP	CGG​AGA​GGT​CGC​GAT​AGT​CAT​GCT​TAT​CAG​ACT​GAT​GTT​GA
BIP	GAT​GAC​AGT​GAC​ATC​CTG​CCT​AGG​CAG​TGT​CTT​AGC​TGG​TTG​T
B2	TGG​CAG​TGT​CTT​AGC​TGG​TTG​T
miR-34 (miRNA B2)	UGG​CAG​UGU​CUU​AGC​UGG​UUG​U
F2	GCT​TAT​CAG​ACT​GAT​GTT​GA
miR-21 (miRNA F2)	UAG​CUU​AUC​AGA​CUG​AUG​UUG​AGC​TTA​TCA​GAC​TGA​TGT​TGA
B1C	GAT​GAC​AGT​GAC​ATC​CTG​CCT
F1C	CGG​AGA​GGT​CGC​GAT​AGT​CA

### 2.2 Reaction set-up and apparatus

pH responsive buffer 25X was made with 1.25 M Potassium Chloride (catalog number P9541, Sigma Aldrich), 0.2 M Magnesium Sulfate (catalog number M7506, Sigma Aldrich), 2.5% Tween-20 (catalog number P9416, Sigma Aldrich), 75 µM Phenol Red (catalog number P3532, Sigma Aldrich), 1 M Guanidine Hydrochloride (catalog number G3272, Sigma Aldrich), 0.75% Triton-X (catalog number T8787, Sigma Aldrich), Water (catalog number W4502, Sigma Aldrich) and 36 mM sodium hydroxide (catalog number BUF-1530, First Base), and was stored at −20°C. Isothermal buffer (catalog number B0537S, New England Biolabs) comprises of 200 mM Tris-HCl pH 8.8, 100 mM Ammonium Sulfate, 500 mM Potassium Chloride, 20 mM Magnesium Sulfate and 1% Tween-20.

LAMP reaction set-up was prepared using the components as shown in Table 2 and real time fluorescence measurement for 20 µL LAMP reactions was performed by Quanstudio5 real-time PCR machine. The fluorescence dye used for detection is EvaGreen (catalog number #31000, Biotium). The TA values are derived using the relative threshold algorithm on QuantStudio software.

**TABLE 2:** Components in a LAMP reaction.

Components	Concentrations
dNTPs (catalog number N0446S, New England Biolabs)	1.4 mM
EvaGreen (catalog number #31000, Biotium)	0.5X
pH responsive buffer 25X (refer to Section 2.2, in-house buffer)/Isothermal buffer 10X (catalog number B0537S, New England Biolabs)	1X
Template Ultramer (Integrated DNA Technologies)	As stated in Section 2.2
Primers (Integrated DNA Technologies)	As stated in Section 2.2
WarmStart Bst 2.0 (catalog number M0538M, New England Biolabs)	6.4 U

For the DNA assay, the concentrations of B2, B1c, F2, F1c and template were 1.6 µM and 4.24 ng respectively while LF and LB were 0.4 µM in the 20 µL reaction unless otherwise stated in text. All reactions were performed at 65°C in a thermocycler for 60 min unless stated otherwise.

For the miRNA assay, the concentrations of B2, B1c, F2, F1c and template were 0.1 µM and 4.24 ng respectively while LF and LB were 0.4 µM in the 20 µL reaction unless otherwise stated in text. All reactions were performed at 60°C for 60 min unless stated otherwise.

For commercial and pH-responsive buffer selectivity tests, the concentrations of B2, F2 and template were 0.3 µM and 4.24 ng respectively while F1C, B1C and LB were 0.1 µM in the 20 µL reaction. All reactions were performed at 60°C for 30 min.

HEK-293 cells were grown in Dulbecco’s Modified Eagle’s Medium (DMEM) (catalog number 12491015, ThermoFisher Scientific) under standard conditions in a humidified incubator at 37°C. Total cell RNA extraction from 200,000 HEK-293 cells was then performed using Direct-Zol RNA Miniprep kit (catalog number R2081, Zymo Research), according to manufacturer instructions.

## 3 Results

### 3.1 Replacing BIP/FIP with two primers enable robust Split-LAMP assay

**FIGURE 1:**
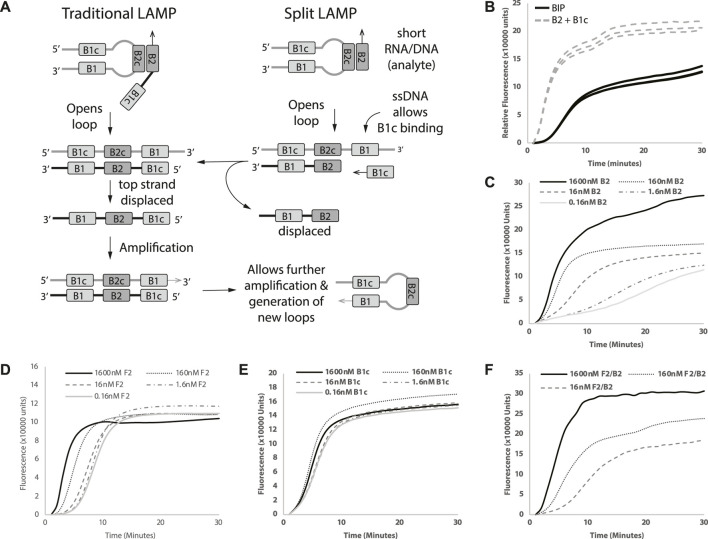
Proof of Split-LAMP concept. **(A)** Illustration of Split-LAMP design *versus* Traditional-LAMP design. **(B)** Replacement of BIP with B2 and B1c. **(C–F)** Decreasing concentrations of B2, F2, B1c and B2/F2 respectively. Curves shows fluorescence (Rn) measured over time (minutes). Reactions were performed without replicates.

BIP is an essential component in forming loop structures that facilitate the efficient amplification of linear target DNA. As discussed above, we sought to split the traditional BIP primer into two separate primers—B2 and B1c (Figure 1A), to enable B2 to be the analyte miRNA sequence. To evaluate the effectiveness of splitting BIP into B2 and B1c, we replaced 1.6 µM BIP with the same concentrations of B2 and B1c in a LAMP reaction. The replacement of BIP with B2 and B1c led to an earlier amplification (Figure 1B). The results from our Split-LAMP reaction showed that B2 and B1c can be used as separate oligonucleotides to replace a single B1P primer.

Since we demonstrated that BIP can be replaced by B2 and B1c, we wanted to see if the time to amplification (TA) was inversely correlated with the concentrations of B2 to identify which primers in Split-LAMP are suitable to be replaced by miRNA analytes. To ensure that Split-LAMP is sensitive to changes in analyte concentrations, we identified primers that show a marked change in reaction fluorescence as their concentration changes. A serial dilution of B2 with constant B1c concentration resulted in correlated increase in TA (Figure 1C) when 1.6 µM of FIP, 0.1 µM of B1c and LB were added in the reactions. An exponential decrease in the concentration of B2 from 1600 to 0.16 nM resulted in longer amplification times. Next, we investigated if a similar effect could be seen with F2 by splitting FIP to F1c and F2, while keeping the other primers constant. In this case, B2 and B1c were used instead of BIP to see if B2, B1c, F2, F1c could be used together.

Similarly, with B2, serial dilutions of F2 concentration increased the TA correspondingly as F2 concentration decreases (Figure 1D). Next, we wanted to see if the LAMP reaction was also sensitive to B1c concentrations. However, reducing the concentrations of B1c showed little to no difference in amplification time (Figure 1E). Hence, we can potentially design LAMP reactions in which endogenous miRNAs will act as B2 and F2 for the Split-LAMP assay.

We wanted to ensure that Split-LAMP functions as an AND gate, requiring the presence of two separate analytes for a single output. To evaluate the functionality of AND gate concept in Split-LAMP, we introduced both F2 and B2 into the same reaction, examining whetehr a single output could be enhanced with a broader spread between the highest and lowest concentrations of B2 and F2, in comparison with individual F2 and B2 analytes. Concomitant decreases of F2 and B2 from 1600nM to 16 nM were found to increase the amplification time more (Figure 1F) than with B2 (Figure 1C) or F2 (Figure 1D) alone. We demonstrated a functional ANDgate concept by showing that TA is dependent on both B2 and F2.

### 3.2 Optimization of concentrations of loop primers in Split-LAMP

**FIGURE 2:**
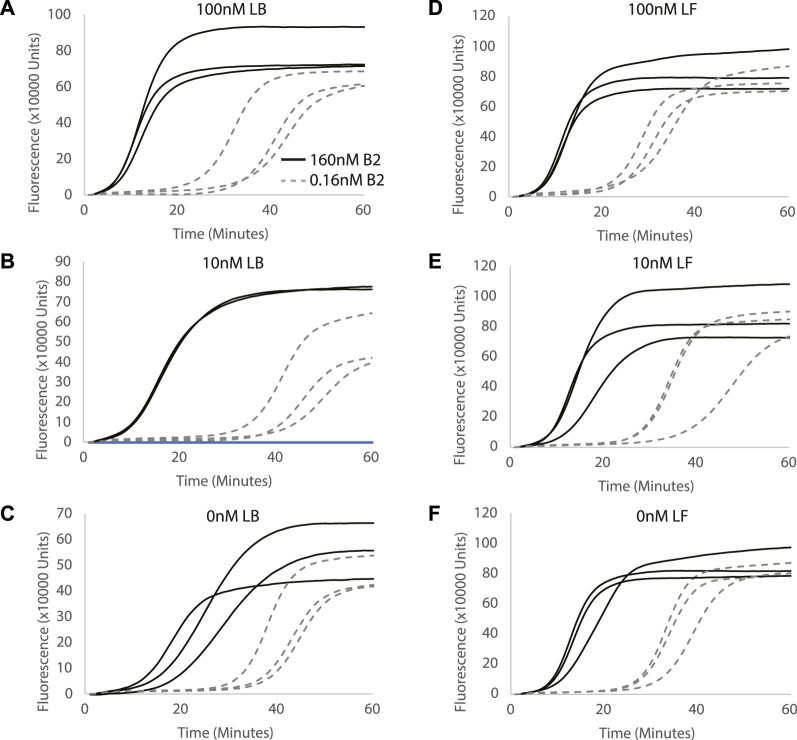
Evaluating the effect of different LB and LF concentrations on miRNA detection. Concentration of LB primer is used as **(A)** 0.1µM, **(B)** 0.01 µM and **(C)** 0 µM. Concentration of LF primer is used as **(D)** 0.1µM, **(E)** 0.01 µM and **(F)** 0 µM. Curves show fluorescence (Rn) measured over time (minutes). All reactions were performed in triplicates.

LF and LB primers were designed to increase branching from the reverse complement loop formed in the reaction and accelerate the Split-LAMP reaction. The concentrations of sequence-specific loop primers, LF and LB, in Split-LAMP were optimized systematically. To investigate the effect of the concentration of LB on target detection, we titrated LB concentration (100, 10, and 0 nM) with 160 and 0.16 nM of B2 in the Split-LAMP assay to determine which gave the largest spread in TA values between these two concentrations (Figures 2A–C). The ΔTA value between 160 and 0.16 nM of B2 increases greatly when LB concentration is increased from 0 to 10 nM LB and remains constant when LB concentration is increased from 10 to 100 nM. We conclude that both LB concentrations of 10 and 100 nM are feasible for use in this assay. 100 nM of LB is selected for further experimentation.

Correspondingly, to investigate the effect of the concentrations of LF on target detection by the Split-LAMP assay, 100, 10, and 0 nM of LF were used to detect 160 and 0.16 nM of F2 in the Split-LAMP assay (Figures 2D–F). We observed that with increasing concentrations of LB used in the LAMP reaction, ΔTA value between 160 and 0.16 nM of B2 decreases. The ΔTA value between 160 and 0.16 nM of B2 and reaction time is the greatest when the concentration of LF primer is decreased to 0 nM. Hence, we conclude that 0 nM of LF is optimal for the Split-LAMP assay.

### 3.3 Quantification of target DNAs

**FIGURE 3:**
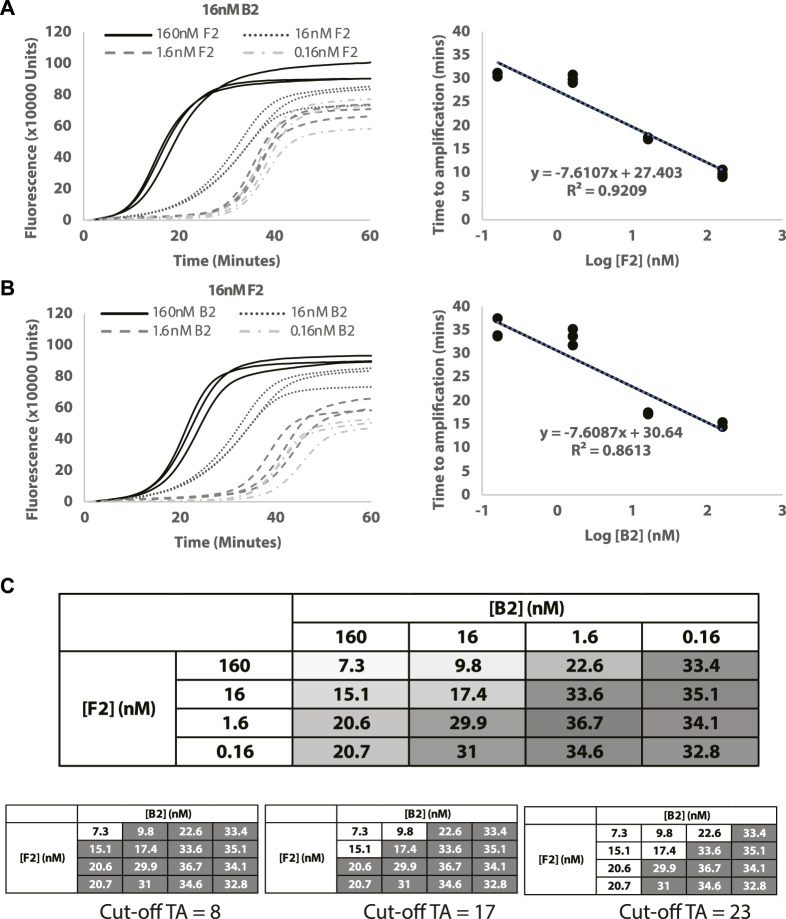
Quantification of target DNAs. **(A)** Decreasing concentrations of F2. **(B)** Decreasing concentrations of B2. Curves show the normalized fluorescence over time (minutes). **(C)** Table showing changes in TA values (ΔTA) measured with different combinations of B2 and F2 concentrations used. The intensity of the colour in the box is a visual representation of the TA value (white = lowest TA; dark gray = highest TA). All reactions were conducted in triplicates.

To assess the potential of Split-LAMP assay for quantitative analysis, reactions with varying B2 and F2 concentrations were performed under optimised LB and LF concentrations from the previous section. The optimised LAMP parameters resulted in increased dynamic ranges for B2 and F2 (Figures 3A, B), showing that Split-LAMP is sensitive to B2 and F2 concentrations in the range of 1.6nM and 160 nM (approximately 1nM–200 nM). The linear relationship between TA values and log concentrations of B2 and F2 (Figures 3A, B) indicates that Split-LAMP is capable of accurately quantifying B2 and F2. The amplification time was measured and used to quantify target DNAs as shown in Figure 3C. A suitable cut-off amplification time can be set such that any amplification before the cut-off marks a readout in which there will be a positive output achieved only when both B2 and F2 are above the desired concentrations. The choice of cut-off times can vary depending on specific diseases. Examples of different cutoff marks resulting in positive output (white box) from different combinations of B2 and F2 concentrations are shown in Figure 3C. Hence, an appropriate amplification time cut-off can be set to estimate the B2 and F2 concentrations.

### 3.4 Quantification of target miRNAs

**FIGURE 4:**
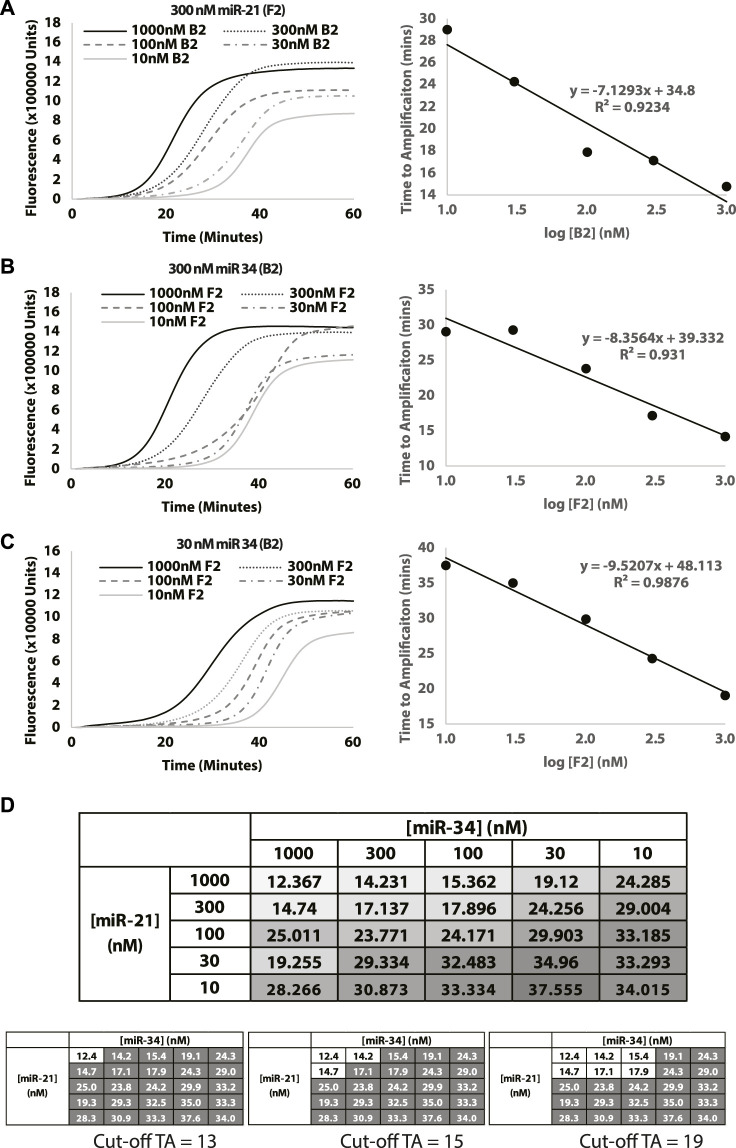
Evaluating the effect of miR-21 and miR-34 concentrations on miRNA detection. Concentration of miR-21 is used as **(A)** 300 nM and concentration of miR-34 is used as **(B)** 300 nM and **(C)** 30 nM. Curves show fluorescence (Rn) measured over time (minutes). **(D)** Table shows change in TA values (ΔTA) measured with different combinations of miR-34 and miR-21 concentrations. The intensity of the colour in the box is a visual representation of the TA value (white = lowest TA; dark gray = highest TA). Additionally, different cutoffs will result in different combinations of the miRNAs that would result in a positive (white box) signal. Gray boxes represent a negative output. Reactions were performed without replicates.

To evaluate the ability of the Split-LAMP assay to quantify miRNA, experiments were performed to detect different concentrations of chemically synthesized miR-34 (B2) and miR-21 (F2) ranging from 10 to 1000 nM. Figures 4A–C display the real-time fluorescence curves generated by varying the concentration of miR-21 (F2) or miR-34 (B2) in the presence of a fixed concentration of the other miRNA listed above. The ΔTA value was recorded and used to monitor the concentrations of miRNA. As shown in the corresponding scatter plot, the ΔTA value is proportional to the concentration of miRNA being varied with relatively good *R* ^2^ values. The full range of amplification curves for different miR-21 and miR-34 concentrations can be found in Supplementary Figure S1. Figure 4D was used to ascertain the logic gate nature of the two inputs and based on shading, clearly demonstrates a diagonal pattern when it comes to the output ΔTA. This allows us to establish that a positive signal can be observed when two miRNAs are present together at the concentration range, enabling simultaneous detection of two miRNAs at the same time. A similar example of selection of cut-off times to determine the range of F2 and B2 concentrations that would result in a positive signal is shown in Figure 4D.

### 3.5 Split-LAMP as a prototype POCT device

The logic gate approach to LAMP enables the co-detection of two miRNAs in a single pot reaction with a potentially colorimetric end-point readout after a specific amplification time, suitable for a point-of-care assay. To investigate the selectivity of the Split-LAMP assay for the target miRNA in an abundance of background cellular RNA, 20uL interference assays were performed using total RNA extracted from 200,000 HEK-293 cells with 0.3 µM of miR-34 (B2) and/or miR-21 (F2) or negative control. Using the same nucleic acid sequences as before, the ΔTA resulting from the presence of both miR-34 and miR-21 in the presence of cellular RNA shows that Split-LAMP allows for specific logic-gate based detection of miR-34 and miR-21 in a complex mixture of RNA.

**FIGURE 5:**
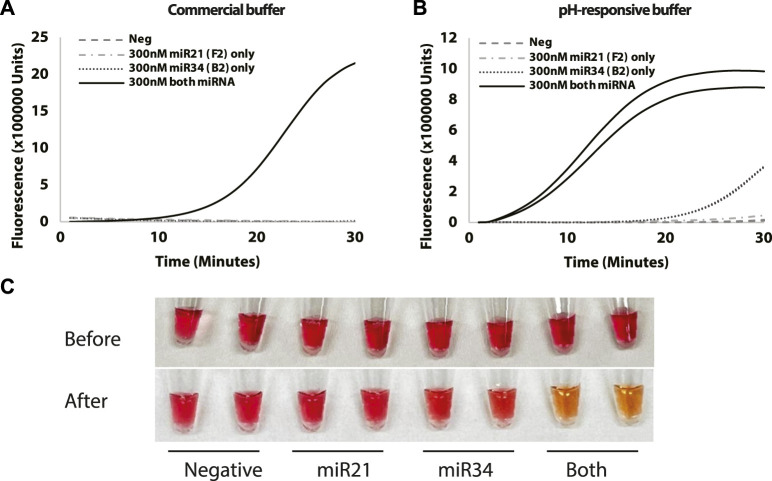
Test for selectivity using **(A)** Commercial buffer and **(B)** pH-responsive buffer with reactions containing miR-34a and/or miR-21 or negative control. **(C)** Images taken at 0 and 30-min timepoint showing colour change of the reaction mixtures. Curves show fluorescence (Rn) measured over time (minutes). All reactions were conducted in duplicates.

pH-responsive buffer contains phenol red indicator and can be used as a means of colorimetric detection (Tanner et al., 2015). Figure 5B shows an increase in fluorescence with the reaction containing miR-34 and miR-21 before the 30 min mark. In particular, the amplification curves suggest that the pH-responsive buffer may be superior to the isothermal buffer in terms of sensitivity as the time to detection is a lot shorter (Figures 5A, B). To investigate whether the colorimetric detection concept is feasible, images were taken at the 0h and 0.5 h timepoint to observe the colour change for the reactions with 0.3 µM of miR-34 and/or miR-21 or negative control. We observed that the colour of reaction mixture changes from pink to orange only with the presence of miRNA-34 and miRNA-21 while other reactions with miRNA-34 or miRNA-21 or negative control remained pink (Figure 5C). This is due to the lowering of background pH caused by a release of protons during amplification. This indicates that an amplification can simply be detected by observing a colour change based on the presence of analytes.

## 4 Discussion

### 4.1 Background

Aberrant expressions of miRNAs are commonly observed in life-threatening illnesses. They serve as powerful biomarkers for clinical diagnosis. Pancreatic cancer, in particular, is highly aggressive and often presents with poor prognostic outcomes due to a lack of symptoms that resulted in late diagnosis (Ferlay et al., 2015; Hayes et al., 2018). Early detection of pancreatic cancer through a combination of biomarkers and POCT is key for increasing survival rate (Hayes et al., 2018). In a recent study, miR-18a and miR-106 have been identified as non-invasive biomarkers for early detection of pancreatic ductal adenocarcinoma, the most lethal type of pancreatic tumor (Xu et al., 2023). Therefore, the development of multiple miRNAs detection-based approaches tailored towards POCT is a significant step towards improving clinical outcomes. One step LAMP is increasingly being utilized as a POCT method, primarily due to its cost-effectiveness, simple colorimetric detection, rapid reaction time of less than an hour and the ability to perform reactions at a constant temperature (Moehling et al., 2021). Compared to traditional methods such as PCR, LAMP does not require complex instruments, making it a cost-effective option to operate. These advantages contribute to the ease of testing and enhance accessibility to patients. However, there is limited availability of LAMP assays that could detect a panel of miRNAs. In this literature, we successfully designed a split LAMP assay that incorporates an ANDgate concept as a POCT method for detecting the presence or absence of two miRNAs by generating a single output with a simple “YES” or “NO” answer.

### 4.2 Split-LAMP mechanism

We have successfully demonstrated the feasibility of splitting FIP and BIP into F2 & F1c and B2 & B1c respectively, to facilitate the replacement of miRNAs with shorter primers. FIP and BIP are chosen as they play a critical role in creating the loop structure from linear double stranded analyte, thereby making the reaction to be dependent on these primers. This innovative design is necessary and has not been attempted previously as replacement of BIP and FIP with miRNAs is hindered by length constraints of these primers. B2 is found to be more effective than B1c in our preliminary tests and is selected as an analyte. In the split miRNA LAMP scheme, B2 causes displacement of the loop which then allows the B1 sequence to be single stranded as B1 is no longer bound within the loop structure. Consequently, B1c primer then binds to the liberated B1 sequence. The amplification from B1c then results in the same structure as that achieved by the BIP primer in traditional LAMP. Our investigations have also proven that the splitting of FIP can be replicated on the other end as LAMP reactions are dependent on F2. Collectively, B2 and F2 then generate the displaced strand, that is, subsequently discarded. The nature of these primers, whether DNA or RNA, does not affect the outcome as both lead to a dead-end amplification rather than a loop formation. As a result, a functional ANDgate is created, where amplification occurs only in the presence of both F2 and B2.

### 4.3 Factors that affect Split-LAMP

Two main factors affecting the amplification time are reaction temperature and concentration of loop primers (LF and LB). The reaction temperature of Split-LAMP affects both the hybridization efficiency of primers and the activity of Bst 2.0 WarmStart DNA polymerase. Conducting the reaction at a lower temperature of 60°C allows for more effective hybridization of miRNAs and DNA primers to their respective complementary sequences on the template while remaining within the ideal temperature range for Bst 2.0 Warmstart DNA polymerase. Loop primers act as a source of loop opening independent of F2 and B2, allowing for amplification by F1C and B1C respectively (Nagamine et al., 2002). pH-responsive buffer was selected for the assay because the colour change of the BRS solution (pink to orange) is more suitable to allow for visual detection of “YES” or “NO” output in POCT contexts. pH responsive buffer is also a cost-effective alternative to state-of-the-art isothermal buffer, as it does not require the use of a reader to detect changes in fluorescence. It is worth noting that theoretically the fluorescence curve of the blank in aforementioned experiments will be a straight line. The blank signal arises gradually, which may originate from un-primed but templated nonspecific DNA synthesis.

### 4.4 Limitations and future applications

The range of detection for Split-LAMP is on the scale of nanomolar concentrations of miRNA in a patient sample. Hence, pre-processing steps may be required to concentrate the sample to allow for detection with the assay. Furthermore, a significant excess of either miRNA-34 or miRNA-21 could potentially compensate for the other, leading to a positive reaction. However, this issue can be mitigated by carefully tuning the expected input within a specific concentration range where neither analyte reaches extreme concentrations through dilution. The current Split-LAMP assay has not been established to differentiate between small nucleotide differences and will be investigated in future studies to further improve specificity for miRNA detection. Additionally, the existing Split-LAMP can be expanded to detect three or more miRNAs by adding more loop structures and corresponding primers to the DNA template. It can also be modified to include OR/NOT gates for various diagnostic applications. These include early detection of ischemic stroke in patients with cancer (miR-205-5p, miR-645, and miR-646) (Bang et al., 2023) and prostate cancer (miR-4289, miR-326, miR-152-3p and miR-98-5p) (Matin et al., 2018).

## 5 Conclusion

In this study, we introduced a new Split-LAMP concept that enables simultaneous detection of two distinct miRNAs. This approach incorporates a rapid colorimetric end-point readout, making it a promising device for POCT. The current Split LAMP system can be further adapted to detect additional miRNAs associated with various diseases. With an urgent demand for miRNA detection to enhance clinical outcomes and patient accessibility, this Split-LAMP holds significant potential as a diagnostic tool for many critical illnesses.

## Data Availability

The original contributions presented in the study are included in the article/Supplementary Material, further inquiries can be directed to the corresponding author.
