# Supplementary material for: Simultaneous and rapid colorimetric detection of distinct miRNAs using Split-LAMP
Source: Front Bioeng Biotechnol. 2023 Nov 2;11:1271297. doi: 10.3389/fbioe.2023.1271297 (PMC10652396; doi:10.3389/fbioe.2023.1271297)
Supplement: Supplementary file 1 [file DataSheet1.DOCX]

Supplementary Material

Simultaneous and rapid colorimetric detection of distinct miRNAs using Split-LAMP

Yi Jing Chua, Steven Poh Chuen Sim, Medha Shridharan, Yiqi Seow

*** Correspondence:** Yiqi Seow: seowy@gis.a-star.edu.sg

# Supplementary Figures and Tables

## Supplementary Figures


**Supplementary Figure 1** Evaluation of the effect of miR-21 and miR-34 RNA concentrations on the time to amplification for the Split-LAMP reactions. The invariant analyte is in the title of each graph with each curve a concentration of the variable analyte. N=1 for each concentration pair.
